# Value of [^18^F]FDG PET/CT radiomic parameters in the context of response to chemotherapy in advanced cervical cancer

**DOI:** 10.1038/s41598-023-35843-9

**Published:** 2023-06-05

**Authors:** Ewa Burchardt, Agnieszka Bos-Liedke, Kamila Serkowska, Paulina Cegla, Adam Piotrowski, Julian Malicki

**Affiliations:** 1 Department of Radiotherapy and Oncological Gynecology, Greater Poland Cancer Center, 61-866 Poznan, Poland; 2 Department of Biomedical Physics, Adam Mickiewicz University, 61-614 Poznan, Poland; 3 Asseco Poland S.A., 44-100 Gliwice, Poland; 4 Department of Nuclear Medicine, Greater Poland Cancer Center, 61-866 Poznan, Poland; 5 Department of Medical Physics, Greater Poland Cancer Center, 61-866 Poznan, Poland; 6 Department of Electroradiology, Poznan University of Medical Science, 61-701 Poznan, Poland; 7 Department of Electroradiology, University of Medical Science Poznan, 61-866 Poznan, Poland

**Keywords:** Cervical cancer, Image processing

## Abstract

The first-order statistical (FOS) and second-order texture analysis on basis of Gray-Level Co-occurence Matrix (GLCM) were obtained to assess metabolic, volumetric, statistical and radiomic parameters of cervical cancer in response to chemotherapy, recurrence and age of patients. The homogeneous group of 83 patients with histologically confirmed IIIC1–IVB stage cervical cancer were analyzed, retrospectively. Before and after chemotherapy, the advancement of the disease and the effectiveness of the therapy, respectively, were established using [^18^F] FDG PET/CT imaging. The statistically significant differences between pre- and post-therapy parameters were observed for SUV_max_, SUV_mean_, TLG, MTV, asphericity (ASP, p = 0.000, Z > 0), entropy (E, p = 0.0000), correlation (COR, p = 0.0007), energy (En, p = 0.000) and homogeneity (H, p = 0.0018). Among the FOS parameters, moderate correlation was observed between pre-treatment coefficient of variation (COV) and patients’ recurrence (R = 0.34, p = 0.001). Among the GLCM textural parameters, moderate positive correlation was observed for post-treatment contrast (C) with the age of patients (R = 0.3, p = 0.0038) and strong and moderate correlation was observed in the case of En and H with chemotherapy response (R = 0.54 and R = 0.46, respectively). All correlations were statistically significant. This study indicates the remarkable importance of pre- and post-treatment [^18^F] FDG PET statistical and textural GLCM parameters according to prediction of recurrence and chemotherapy response of cervical cancer patients.

## Introduction

Cervical cancer is the eight most common malignant tumor in the world and ranks fourth in the female population^1^. In 2020, in as many as 23 countries around the world, cervical cancer was the most frequently diagnosed cancer in women, and in 36 countries it was the leading cause of death among all cancers. According to GLOBOCAN data, in the world population in 2020 there were over 604,000 new cases and over 342,000 deaths caused by cervical cancer. Compared to 2012, there was a noticeable increase in both the incidence and deaths due to cervical cancer^2^. Standard therapeutic approach for locally advanced cervical cancer (LACC) is chemoradiotherapy (CRT). The International Federation of Gynecology and Obstetrics (FIGO) introduced in 2018 a new cervical cancer classification because metastases in paraaortic lymph nodes (PALN) significantly influence patients' survival. However, the extended field radiotherapy, as the recommended first-line treatment, seems to be insufficient as the median patients’ survival time is 33 months and 40% of patients develop distant metastases^3^. The use of systemic neoadjuvant therapy, followed by radiotherapy, may be beneficial for improving the prognosis of these patients. The ongoing trial NCT01566240 aims to answer this emerging question.

Among the diagnostic techniques, positron emission tomography (PET) in combination with computed tomography (CT) has become the most informative one. Application of 2-deoxy-2-[^18^F]fluoro-D-glucose, (2-[^18^F]FDG) PET/CT is well-established in determining the advancement of cervical cancer. Further, positive PALN in [^18^F]FDG PET/CT was shown as the most significant prognostic factor of progression-free survival (PFS)^4^. [^18^F]FDG PET/CT examination, in addition to information on the localization of lesions, allows the assessment of biological and metabolic features. However, imaging can also provide a more comprehensive view of the tumor in its entirety via radiomics. This field of medical study develops mathematical methods of analyzing tomographic images to extract certain data that can be used as prognostic features to predict the progression of neoplastic diseases and their response to treatment. Textures describe the spatial distribution of patterns in an image. They are believed to be a rich source of information about internal structure and surface and are used to quantify the spatial relationships contained in an image. Each of the elements consisting of pixels or voxels creates a texture and has its own characteristic parameters: size, shape, brightness. The arrangement of the groups with the same values of the listed features influences the local properties of the texture. Textures are second-order features and can be calculated from the matrix. GLCM defines the distribution of certain combinations of the intensity of gray levels of adjacent pixels or voxels along with one of the directions in the image. Although radiomics has shown promise to be an imaging biomarker of different tumors in clinicals^5^, not every research has been yet translated into practice. However, there is an effort in the scientific community to bring together the large number of meaningful quantitative features from medical images in order to translate them into an essential clinical decision-making tool. This is the case already in breast cancer. Fanizzi et al. proposed model for prediction of the microcalcification clusters at mammograms with the sensibility = 91.78%^6^ Similarly in lung cancer, quantitative analyses of low-dose computed tomography lung cancer screening images could predict nodules that would become cancerous in 1 year hence with accuracy of 80%^7^. The next promising direction would be the use of deep learning models, as they can extract features automatically using Artificial Intelligence (AI)^8^. However, the use of such powerful techniques require a large patient database to achieve accurate classification, fast computing systems and large memory resources^9^. For that reason, textural analysis is easier to implement and run in clinical science.

The aim of this work is a first-ordered statistical and second-ordered textural analysis to obtain prognostic metabolic parameters and radiomic features of the cervical cancer tumor in response to chemotherapy. Second-ordered textural analysis was done using GLCM.

## Results

All metabolic, volumetric, textural and clinical parameters analyzed in this work, except the age of patients, show non-normal distribution according to the Shapiro–Wilk test. Non-parametric Wilcoxon test was used to compare the pre- and post-chemotherapy features.

The statistically significant differences were observed for metabolic and volumetric parameters, namely SUV_max_, SUV_mean_, TLG, MTV and ASP (p = 0.000) (Z > 0) as well as for first-order PET (E, p = 0.000) and second-order PET (COR, E_n_ and H with p = 0.0007, p = 0.000, p = 0.0018, respectively).

Among first-order parameters low correlation was found between response to chemotherapy and pre- and post-treatment Shannon entropy calculated from PET data (R = −0.28, R = −0.27, respectively, p < 0.05), post-treatment coefficient of variation (R = −0.27, p = 0.01), and kurtosis (R = 0.25, p = 0.02). Recurrence and pre-treatment COV were observed to have a moderate positive correlation (R = 0.34, p = 0.001) while between recurrence and E the correlation was low and negative (R = −0.22, p = 0.047).

Among second-order textural parameters pre-treatment contrast and energy showed moderate negative (R = −0.31, p = 0.0038) and low negative (R = −0.22, p = 0.044) correlation with the age of patients. Chemotherapy response strongly correlates with post-treatment En (R = 0.54, p = 0.00) and moderately with H (R = 0.46, p = 0.00), whereas it showed low negative (R = −0.23, p = 0.035) and positive (R = 0.29, p = 0.0063) correlation to C and COR respectively.

All correlations between metabolic, volumetric, textural and clinical parameters before and after chemotherapy are shown in Supplementary materials.

## Discussion

The following study demonstrated that neoadjuvant chemotherapy (NACT) in patients with cervical cancer of stage IIIC1–IVB significantly affects some of the radiomic and metabolic parameters obtained from positron emission tomography. Presented results reflect the influence of applied chemotherapy on a primary tumor. There is a significant difference in some metabolic (SUV_max_, SUV_mean_, TLG), volumetric (MTV, ASP) and radiomic (E, COR, E_n_, H) features. This corresponds to many studies that have already presented analysis of [^18^F]FDG PET/CT metabolic parameters as a prognostic factor in cervical cancer patients treated with different techniques. Already in 2016 Herrera et al. has shown that pre-treatment tumor glycolytic volume derived from MTV and SUV_mean_ as well as metabolic activity of [^18^F]FDG–positive disease provide useful information for survival and recurrence in patients with CC treated with chemoradiotherapy^10^. Recently, Wang et al.^11^ presented pre-treatment TLG to be an important prognostic biomarker in 125 LACC patients and showed that pre-treatment cervical and lymph-node [^18^F]FDG PET/CT metabolic parameters are associated with survival outcome. However, further studies were required to assess whether overall survival can be determined with metabolic factors only. In one of the latest analyses, Pedraza, et al. showed in the cohort of 116 patients with FIGO stage IB2-IVA LACC treated by primary radiochemotherapy that higher TLG, MTV and sphericity were significantly associated with prognosis for OS in patients with LACC^12^. However, predicting the OS is not the only parameter of interest for authors. There are studies on the use of metabolic parameters as indicators for predicting treatment response in cervical cancer^13,14^. Higher SUV_max_, MTV and TLG have been presented by Liu, et al. in 240 patients with stage IA2-IIA2 ECSC (early-stage uterine cervical squamous cancer) to be associated with larger tumor sizes and deeper stromal invasion. SUV_max_ and TLG appeared to be independent predictors for positive- and intermediate-risk status, but only MTV was a significant indicator. Rufini, et al. enrolled 88 patients with LACC where 40 patients had complete response (CR, pR0), 48 had partial response (PR) with baseline, early and final [^18^F]FDG PET/CT performed before, during and after neoadjuvant chemo-radiotherapy. PR had significantly higher SUV_max_ and SUV_mean_ than CR, whether early MTV and TLG were significantly higher in PR than in CR. Final SUV_max_, SUV_mean_ and TLG were significantly higher in partial responders than in complete responders. Similarly our results showed the statistically significant differences observed for SUV_max_, SUV_mean_, TLG, MTV and ASP, confirming that chemotherapy had a positive effect on reducing metabolism of the cancer lesion.

Although the analysis of [^18^F]FDG PET/CT metabolic factors does not provide unequivocal answers as to their prognostic and predictive significance, their combination with radiomic features enables more accurate determination of the patient's results after chemotherapy.

Except basic [^18^F]FDG PET/CT analysis including metabolic and volumetric parameters, radiomics started to be a major factor for improving early detection, predicting disease progression and assessing treatment response. Mathematical analyses of diagnostic images allow us to see what is invisible to the human eye. So far, most studies have shown the additional value of radiomics to clinical factors. Ferreira et al. tested the feasibility of [^18^F]FDG PET radiomic features combined with clinical information in predicting disease-free survival (DFS) in patients with cervical cancer treated with radiochemotherapy. The most significant predictors of DFS in uni- and multivariate analysis were textural, matrix-based, and intensity histogram^15^. Kidd et al. in 2014^16^ reported that the rates of [^18^F]FDG PET SUV_max_ and MTV decline during chemoradiotherapy progress. In our case, the Wilcoxon test pointed out various parameters that statistically change after therapy, decreasing the glucose metabolic capabilities of the primary cervical cancer. Similar to Kidd et al. SUV_max_, SUV_mean_, TLG, MTV decreased after chemotherapy in the presented study. Moreover, in our study, the group of parameters that changed after chemotherapy are also ASP, E (correlated strongly with TLG and MTV) and second-order textural COR, entropy and homogeneity, that have not been reported in terms of chemotherapy so far. Although in the Wilcoxon test statistical differences appeared between various pre- and post-treatment parameters, only few of them turned out to be potential prognostic features. Fiz et al. presented in a comprehensive review that changes in the level of entropy and homogeneity after chemotherapy correlate with radiological tumor response in liver metastases^17^. In esophagus cancer, a decline in the mean SUV, pre-therapy skewness, and post-therapy homogeneity were predictive characteristics of neoadjuvant chemoradiation therapy response in pathological specimens^18^. Our results directly show that post-chemotherapy En is strongly and H moderately correlated with chemotherapy response and both, higher energy and homogeneity parameters, are observed for responders. Both those parameters are associated with gray level distribution—remaining tumors of the responders have a constant form with smaller gray tone differences in pair elements. The increase of both these parameters with response to chemotherapy is explained by achieving a more homogenous image with lower gray level. Since the cohort consists of patients who received in great majority 6 cycles of chemotherapy, it was not possible to check if the result is anyhow connected to this fact. Furthermore, 93% of the patients included in our study received paclitaxel + cisplatin and it was not possible to distinguish whether the type of chemotherapy is related to the achieved results. For future work, it would be interesting to repeat the analysis for varied in terms of the treatment (cycles, type) received group of patients.

The well-known CC prognostic factors are FIGO stage, LN status and histopathological type. As PET/CT is a gold standard in staging, a radiomic signature related to response to treatment may potentially reflect a tissue phenotype associated with specific biology leading to better risk stratification of patients^19^. In the presented research, there is a lack of correlation between radiomic parameters and FIGO, which results from the homogeneity in the CC stage of the study group.

In our study, COV and recurrence were observed to have a moderate positive correlation, while recurrence and entropy were observed to correlate negatively. Multivariate analysis performed by Lucia et al. identified gray-level non-uniformity (GLNU) from GLRLM in PET as an independent prognostic factor in LACC treated with RCT. Together with entropy from GLCM in ADC maps from MR, they showed an accuracy of 94% in predicting recurrence^20^. The study developing radiomics signatures based on the fused PET/CT showed different subregions of the LACC tumor significantly associated with PFS and OS^21^. Rauzé et al.^22^ investigated the aspect of cervical cancer prediction by textural features. In this study on 79 subjects, local recurrence in patients has been identified by i.a. SUV_mean_, SUV_max_, SUV_peak_, MTV, TLG and entropy. Out of those features, only entropy correlated with recurrence in our case, but the article indicates the strong impact on the PET device used for the patients imaging on the achieved parameters. Furthermore, the correlations between SUV_max_, MTV and TLG as well as first- and second-order textural features are not neglectable.

Radiomic features can be investigated also in magnetic resonance imaging (MRI) images to obtain the best possible view of predictive factors. In cervical cancer, there are some reports investigating radiomic features from MRI as a potential tool for predicting response to therapy. Sun et al. presented in the multicenter study a treatment group receiving NACT and found intratumoral and peritumoral regions of two radiomic sequences with the best predictive power^23^. The microenvironment of peritumoral regions play an important role in immune response. This is also the specific area of interest, where the microscopic tumor load is found and during brachytherapy treatment is called the “high risk Clinical Tumor Volume” (hrCTV). It would be interesting to analyze radiomic features from MRI at time of brachytherapy application. It was shown in other types of cancer, that the radiomic signature of peritumoral tissue also plays an important role and could be predictive for recurrence^24^. In a study of Ciolina et al. the kurtosis extracted from the primary tumor of baseline MR, showed a significantly higher value in responding patients and was indicated as a predictor of tumor response to platinum-based neoadjuvant chemotherapy^25^. Ytre-Hauge et al. in their prospective evaluation of 180 patients in MR demonstrated that high kurtosis in post-contrast T1-WI was a good predictor of reduced recurrence as well as progression-free survival (HR 1.5, p < 0.001). Additionally, high tumor entropy in MR independently predicted deep myometrial invasion^26^.

Tumor heterogeneity can be defined by inter-tumor and intra-tumor heterogeneity. The first one refers to primary cancer diversity between patients whose altered geno- and phenotypes were induced by various factors (e.g. environmental, etiological). Intra-tumor heterogeneity describes genomic and biological variations within a tumor lesion^27^. Many articles, however, discuss tumor heterogeneity as one phenomenon without deeper characteristics of the feature. Biologically, coefficient of variation indicates tumor heterogeneity. Statistically, it is a normalized measurement of the dispersion of the SUV inside the VOI, so dispersion of the glucose metabolism in the investigated volume. Grabinska et al. showed that in gastric cancer patients increased COV is associated with poor tumor differentiation^28^. Although, the usefulness of coefficient of variation to assess histopathologic response early in therapy and after therapy was presented in locally advanced rectal cancer^29^. Up to our knowledge there was no published evidence of COV correlation to chemotherapy response and recurrence in advanced stages of cervical cancer. Chung et al.^30^ showed such a correlation only for an early stage (IB–IIA) uterine cervical cancer before surgery. Results of our study directly show that the pre-chemotherapy COV is able to predict the recurrence of the patients with cervical patients in stage IIIC1–IVB. There is a great advantage of using the same imaging modality in the post-chemotherapy setting as in the pre-chemotherapy setting. It enables the direct comparison of examined features. As we know, the [^18^F]FDG PET/CT is the best imaging modality for staging in cervical cancer, especially considering lymph nodes involvement and distant metastases, as in this analysis. Different methods used in radiomics aim to obtain prognostic features in cancer. In one of the first studies of tumor heterogeneity characterized by textural features, changes during radiochemotherapy differentiated patients with complete response from partial and non-responders^31^. Different textural features, such as high gray-level run emphasis HGRE determined pelvic recurrence^32^, pretreatment gray-level nonuniformity GLNU (GLRLM) textural analysis, intra-treatment decline of run length nonuniformity < 55% and the decline of TLG < 60% during radiochemotherapy were associated with poor OS^33^. In multivariate analysis done by Chen et al. a low HGRE (high gray-level run emphasis) was a prognostic factor for low OS and inferior PFS^32^.

There are some limitations. The retrospective design of this analysis might have affected population selection. Moreover, it is suggested that the radiomics study should incorporate an enormous data set. We are aware that the number of patients in this study is limited, but it should be considered a part of the extensive worldwide analysis and one of many steps in this process. The main obstacle of radiomics is analysis of non-uniform images, differing approach to data processing and repeatability across studies^33,34^. In the future, it could be solved with the use of AI and Convolutional Neural Network where the feature extraction and classification steps are unified. The convolutional neural network model does not demand explicitly generated features as input because feature representations are discovered during training. A very interesting, non-invasive way of using the Deep learning approach was presented in the work of Brinker et al.^35^. They developed a digital biomarker that could predict lymph node metastasis from digitized hematoxylin and eosin stained pathology slides of primary melanoma tumors. It seems to be a discovery which application in clinical practice is very promising at the step of diagnosis.

Capturing non-visible features responsible for a worse prognosis implies a more aggressive phenotype and stratifies these patients into two subgroups: high-risk and low-risk. It could potentially be beneficial in preparing the treatment strategy. The presented CC patients underwent first-line chemotherapy, followed, after second [^18^F]FDG PET/CT, by radiotherapy. Prognosis evaluation in advance would significantly improve the radiotherapy planning by enhancing the treatment protocol in patients with the poorer outcome of cervical cancer. In the future, radiomics may be used together with well-known clinical and histological metrics (age, stage, histologic type, histologic grade, stromal invasion of the cervix, Lymphovascular Space Invasion, tumor size) as part of a nomogram of features.

## Conclusion

Cervical cancer is a relevant female problem worldwide. According to our results [^18^F] FDG PET textural parameters before and after chemotherapy correlate to chemotherapy response and recurrence to varying degrees. Taking into account the strength of correlations of diverse parameters, there exists a prospective significance to coefficient of variation in predicting recurrence on the basis of pre-treatment [^18^F] FDG PET image, and second-order textural GLCM features, namely energy and homogeneity, in predicting chemotherapy response based on post-treatment [^18^F]FDG PET image.

## Methods

### Patients and inclusion criteria

A retrospective analysis was performed on 83 patients with histologically confirmed cervical cancer of IIIC1–IVB stages^36,37^ according to the FIGO staging system. Patients included in the analysis were treated using chemotherapy in the time range between October 2012 and January 2021 in the regional Oncological Cancer Center in Poznan (Poland). Before and after therapy the advance of the disease and the effectiveness of the therapy were estimated using [^18^F]FDG PET/CT imaging. The post-treatment [^18^F]FDG PET/CT was performed 4 weeks after the last chemotherapy administration. The inclusion criterion covered the appropriate FIGO stage (IIIC1—in this study two cases, IIIC2 or IVB) and availability to all necessary medical information (e.g. chemotherapy response, recurrence). Patients were homogenous in terms of received number of chemotherapy cycles—83% of patients received 6 cycles of chemotherapy, 8%—5 cycles, 6%—4 cycles and remaining two patients 3 and 7 cycles. 93% of the patients received paclitaxel 135 mg/m^2^ + cisplatin 75 mg/m^2^ chemotherapy regimen every 3 weeks (77 patients), three patients paclitaxel + carboplatin, and in two cases paclitaxel + carboplatin was followed by bevacizumab and in one case patient received etoposide + cisplatin. Relevant information about the patients characteristics is given in Table 1.

**Table 1:** Patients characteristics.

Variable	Value
Median age (range)	55 (24–76)
FIGO	
IIIC1	2
IIIC2	52
IVB	29
Histological type	
Squamous cell carcinoma	72
Adenocarcinoma	10
Adenosquamous carcinoma	1
Recurrence	
None	45
Local	13
Distant	25
Type of chemotherapy	
Paclitaxel + cisplatin	77
Paclitaxel + carboplatin	3
Paclitaxel + carboplatin + bevacizumab	2
Etoposide + cisplatin	1

The research has been performed in accordance with the Bioethical Committee guidelines and the Declaration of Helsinki.

### Informed consent statement

The need for ethics approval and informed consent was waived by an institutional review board, namely Bioethics Committee Poznan University of Medical Sciences, and was deemed unnecessary according to national regulations.

### Image acquisition

PET and low-dose CT images were performed simultaneously using Gemini TF PET-CT scanner (Phillips, Cleveland, USA). Pre- and post-treatment parameters of the imaging process were identical. Patients fastened at least 6 h before administration of [^18^F]FDG. Serum glucose concentration was measured right before the injection of the radiopharmaceutical. Acquisition started ca. 60 min after intravenous injection of [^18^F]FDG. The mean activity of the radiotracer was 3.7 MBq/kg of body weight. The scans were scattered and randomly corrected and reconstructed using OSEM reconstruction (MTX = 256 × 256). The field of view (FOV) was 18 cm with a slice thickness of 5 mm. After administration of [^18^F]FDG, patients were resting in a darkened room at room temperature.

### Analyzed parameters

Since the areas of distant nodes were estimated as too small (few voxels in PET/CT image), the analysis was performed only according to primary tumor in both pre- and post-treatment [^18^F]FDG PET/CT images. Fusion of images, and fixed threshold-based (Th = 35%) delineation of volume tumor was evaluated using ROVER® (version 3.0.50, ABX GmbH, Radeberg, Germany^38,39^). Metabolic and volumetric parameters of PET image were assessed.

Basic analysis of [^18^F]FDG PET parameters included metabolic and volumetric parameters: standardized uptake values (SUVs), metabolic tumor volume (MTV), total lesion glycolysis (TLG) and asphericity. Inter-tumoral heterogeneity was assessed by COV and intra-tumoral heterogeneity by FOS parameters related to histogram features—skewness (SKE), kurtosis (K), empirical entropy. SKE, K and E parameters were calculated using the “e1071” and “entropy” R packages, respectively.

Radiomic second-ordered textural parameters, namely contrast, correlation, energy and homogeneity were evaluated in MATLAB software (version R2017a, The MathWorks, Inc., Natick, MA, USA^40^) using a gray-level co-occurrence matrix.

SUV referred to a ratio of the measured activity concentration accumulated in the delineated volume of tissue $\left[\frac{Bq}{mL}\right]$ to the activity injected to the patient $\left[Bq\right]$ and normalized by body weight $\left[g\right]$^41^.

$$SUV=\frac{measured\,activity\,concentration\,in\,the\,tissue}{Activity\,injected\,to\,the\,patient}\times body\,weight$$

SUVs parameters included SUV_max_ and SUV_mean_ (the average uptake in the tumor). MTV was a summed volume of metabolically active tumor segmented using appropriate threshold-based method given in [cm^3^]. TLG was calculated as a product of SUV_mean_ and MTV. On the basis of PET image, the asphericity of the tumor was defined as quantitative deviation from circular metabolic volume of the tumor^42^

$$ASP = \sqrt[3]{\frac{1}{36\pi }\frac{{S}^{3}}{{V}^{2}}}-1$$

where *V* is the volume and *S* the surface of the* MTV.*

Kurtosis, skewness^43^ and empirical Shannon entropy^44^ were defined as shown in Table 2.

**Table 2:** Definitions of the kurtosis, skewness and Shannon entropy.

Parameter	Equation	Description
Contrast	$C={\sum }_{i,j}{\left|i-j\right|}^{2}\bullet GLCM(i,j)$	*i*,*j*—values of the neighboring voxels; *GLCM*(*i*,*j*)—*i,j *element of the GLCM; ${\mu }_{i}$, ${\mu }_{j}$—mean value of *i *or *j* column; ${\sigma }_{i}{\sigma }_{j}$—variation of *i* or *j *column
Correlation	$COR={\sum }_{i,j}\frac{(i-{\mu }_{i})(j-{\mu }_{j})\bullet GLCM(i,j)}{{\sigma }_{i}{\sigma }_{j}}$	
Energy	$En={\sum }_{i,j}{GLCM(i,j)}^{2}$	
Homogeneity	$H={\sum }_{i,j,}\frac{GLCM(i,j)}{1+\left|i-j\right|}$	

Kurtosis, skewness and entropy are pure statistical parameters. K measures the shape of the probability distribution. It gives information about the flatness of the histogram and how the data distribution differs from the normal distribution. SKE represents the asymmetry of the probability distribution of histogram pattern. The entropy provides statistical information about the irregularities in the histogram and describes the variation of the parameters of interest distribution. SKE, K and E parameters were calculated using the “e1071” and “entropy” R packages, respectively. COV was calculated from VOI as the ratio of the standard deviation to the SUV_mean_.

Gray-Level Co-occurence Matrix is a basic source of information about the texture of an image. GLCM describes the position of the neighboring voxels with a certain gray level alongside the chosen direction in a tumor model, which results in calculating the intensity distribution. To evaluate the treatment response of cervical cancer patients, contrast, correlation, energy and homogeneity were analyzed^45^. Contrast (variance, inertia) determines the local gray level differences between a voxel and its neighborhood over the entire GLCM. Correlation describes linear dependency of gray-level values. Energy (uniformity, second angular moment) gives information about uniformity of voxel pairs within GLCM. Finally, homogeneity is a measure of how close the elements of GLCM are arranged to the diagonal. Equations used for all textural parameters are shown in Table 3.

**Table 3:** GLCM textural parameters.

Parameter	Equation	Description
Kurtosis	$K=\frac{n(n+1)}{(n-1)(n-2)(n-3)}\frac{{\sum }_{i=1}^{n}{({x}_{i}-{x}_{mean})}^{4}}{{SD}^{4}}-3\frac{{(n-1)}^{2}}{(n-2)(n-3)}$	*x—*a discrete random number, $x=\left\{{x}_{i},\dots ,{x}_{n}\right\};$* n—*the number of voxel (non-missing observations); *SD—*standard deviation; $p({x}_{i})$*—*probability of the event ${x}_{i} \left(p\in \left[\mathrm{0,1}\right]\right)$in the data set. In case of PET and CT images event ${x}_{i}$ is related to SUV value and intensity of the voxel in Hounsfield scale
Skewness	$SKE=\frac{n}{(n-1)(n-2)} \frac{{\sum }_{i=1}^{n}{({x}_{i}-{x}_{mean})}^{3}}{{SD}^{3}}$	
Entropy	$E=-{\sum }_{i=1}^{n}p\left({x}_{i}\right){log}_{2}[p\left({x}_{i}\right)]$	

Analyzed textural parameters were checked versus metabolic, volumetric and statistical PET parameters, and then versus clinical features such as age of patients, FIGO stage, treatment response and recurrence.

### Statistical analysis

Statistical analysis was performed using the Statistica® (version 13.3.0, TIBCO Software Inc, Palo Alto, CA, USA^46^) program. Normal distribution of the data was checked using the W Shapiro–Wilk test. The *p*-value less than 0.05 was considered as significant. Non-parametric Wilcoxon test was used to investigate the influence of chemotherapy on metabolic, volumetric, statistical and textural parameters (p < 0.005). Further, the R Spearman test was used to consider the correlation between individual textural features versus metabolic, volumetric and clinical parameters. A full correlation was assumed with values ranged from $0.9\le R<1$, very strong correlation $0.7 \le R<0.9$, strong correlation $0.5\le R<0.7$, moderate correlation $0.3\le R<0.5$ and low correlation was found in values ranged from $0<R<0.3$. Level of significance was 0.005. Finally, the prediction of long-term survival by all metabolic, volumetric, statistical and textural parameters was examined using the U-Mann–Whitney test.

## Supplementary Information

Supplementary Table 1.

## Data Availability

The datasets used and/or analysed during the current study available from the corresponding author on reasonable request.
