# Supplementary material for: Value of [18F]FDG PET/CT radiomic parameters in the context of response to chemotherapy in advanced cervical cancer
Source: Sci Rep. 2023 Jun 5;13:9092. doi: 10.1038/s41598-023-35843-9 (PMC10241798; doi:10.1038/s41598-023-35843-9)

**Value of [^18^F]FDG PET/CT radiomic parameters in the context of response to chemotherapy in cervical cancer.**

Ewa Burchardt^1,7^, Agnieszka Bos-Liedke ^2*^, Kamila Serkowska ^3^, Paulina Cegla ^4^ , Adam Piotrowski ^2^, Julian Malicki ^5,6^

1. Department of Radiotherapy and Oncological Gynecology, Greater Poland Cancer Center, 61-866 Poznan, Poland,
2. Department of Biomedical Physics, Adam Mickiewicz University, 61-614 Poznan, Poland
3. Asseco Poland S.A., 44-100 Gliwice, Poland
4. Department of Nuclear Medicine, Greater Poland Cancer Center, 61-866 Poznan, Poland
5. Department of Medical Physics, Greater Poland Cancer Center, 61-866 Poznan, Poland
6. Department of Electroradiology, Poznan University of Medical Science, 61-701 Poznan, Poland
7. , Department of Electroradiology, University of Medical Science Poznan, 61-866 Poznan, Poland

*Table 1. Correlations between all parameters before (A) and after (B) chemotherapy. Values marked with bold are statistically significant (p<0.005)*


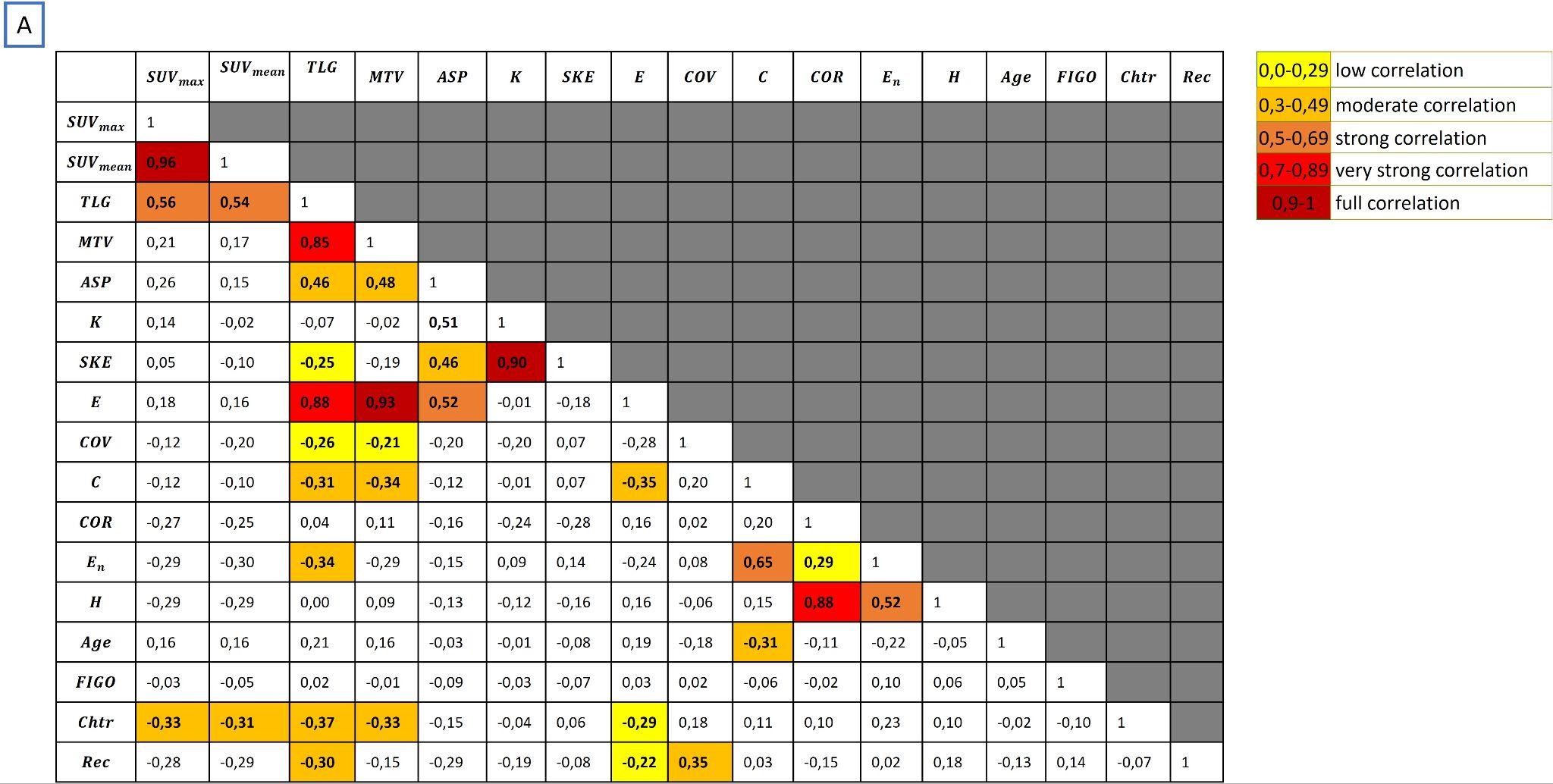


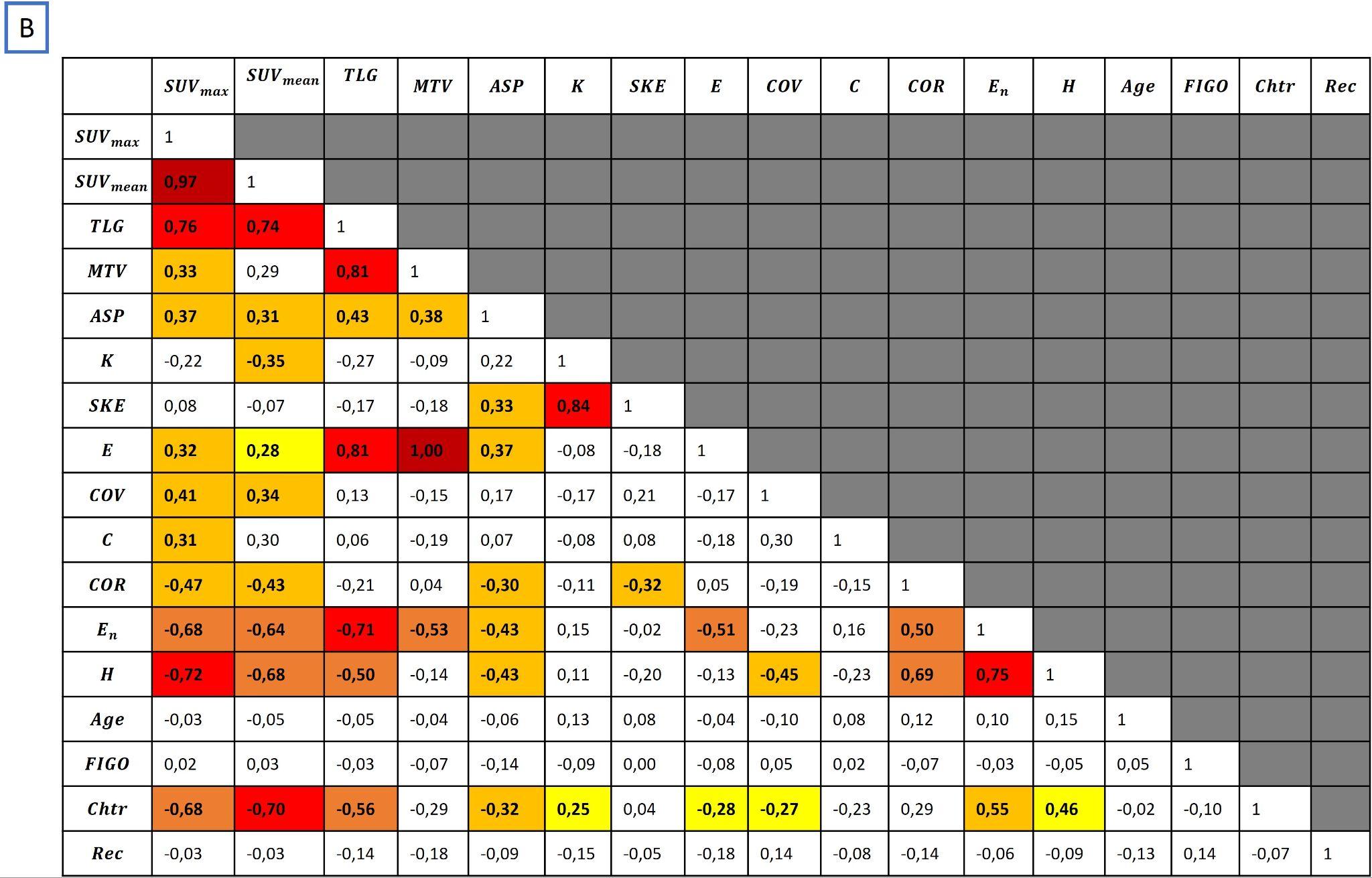

Supplement: Supplementary file 1 — Supplementary Table 1. [file 41598_2023_35843_MOESM1_ESM.docx]
